# Supplementary figures and images for: Dual functioning by the PhoR sensor is a key determinant to Mycobacterium tuberculosis virulence
Source: PLoS Genet. 2023 Dec 15;19(12):e1011070. doi: 10.1371/journal.pgen.1011070 (PMC10723718; doi:10.1371/journal.pgen.1011070)

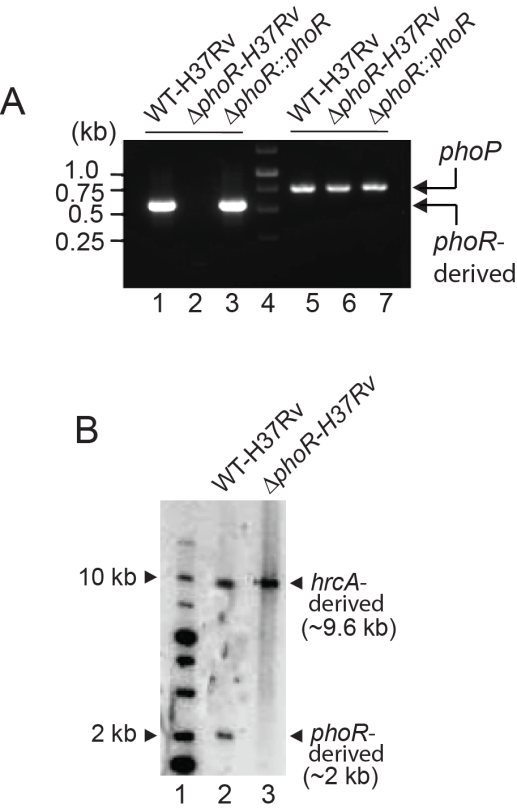

Supplement: S1 Fig — (A) Gene-specific PCR reactions utilized genomic DNA of WT-H37Rv and ΔphoR-H37Rv and a pair of phoR-specific primers (FPphoRInt/RPphoRInt), and products were resolved on agarose gel. Although WT-H37Rv yields a ~0.6 kb- phoR-specific product (lane 1), genomic DNA of ΔphoR-H37Rv fails to yield a phoR- specific amplicon (lane 2). However, the complemented mutant (lane 3) harbouring a copy of phoR shows the presence of the specific amplicon; lane 4, DNA molecular weight marker. As a positive control, phoP-specific amplicon was present in the three strains (lanes 5–7). (B) Southern blot analyses using genomic DNA of WT-H37Rv showed two specific bands when probed with end-labelled hrcA and phoR-specific probes (approximately 9.6- and 2-kb, respectively). However, ΔphoR-H37Rv genomic DNA, using identical probes could detect hrcA-specific band (~9.6 -kb), but not the phoR-specific product. (TIF) [file pgen.1011070.s001.tif]

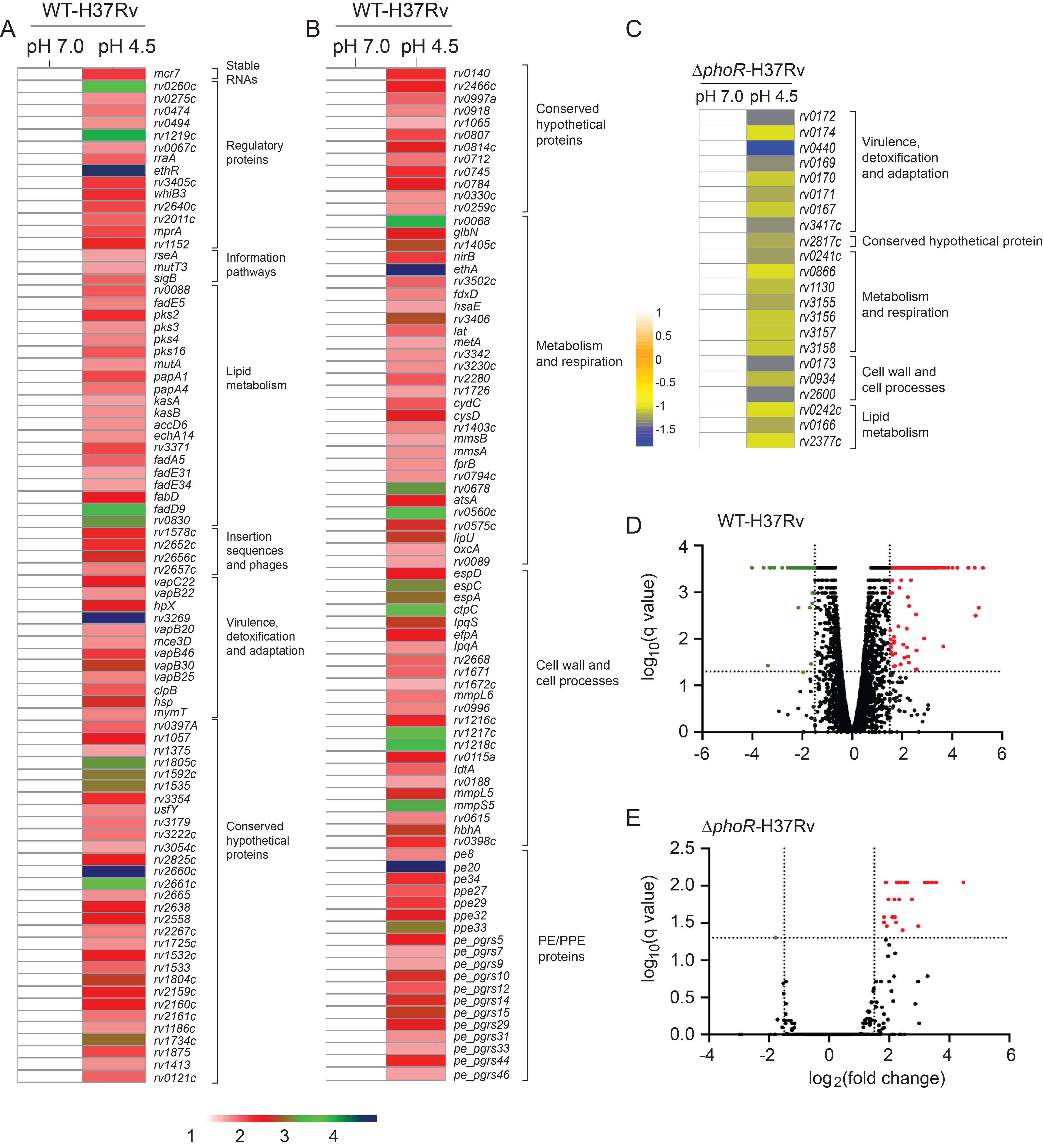

Supplement: S2 Fig — (A-B) RNA-sequencing derived heat-map showing 167 significantly activated acidic pH-inducible genes of WT-H37Rv (>1.5 -fold, p< 0.05, q<0.05) grown under low pH (pH 4.5) relative to normal conditions (pH 7.0) of growth. (C) Expression profile of acidic pH-dependent downregulated mycobacterial genes. RNA-sequencing derived heat-map showing ~ 22 low pH-dependent down-regulated genes in ΔphoR-H37Rv (>1.5 fold; p< 0.05) grown under low pH (pH 4.5) conditions compared to normal conditions (pH 7.0) of growth. The data represent average of biological replicates, and list acidic pH-dependent significantly downregulated genes of the mutant. (D-E) The Volcano plots display differential expression of genes (> 1.5- fold, p< 0.05, q<0.05) grown under low pH (pH 4.5) relative to normal conditions (pH 7.0) of growth in case of (D) WT-H37Rv and (E) ΔphoR-H37Rv, respectively. In these plots, each dot represents expression level of a gene; while red and green dots indicate statistical significance, black dots represent lack of statistical significance. Of the differentially expressed genes, 31 genes were significantly up-regulated (Fig 3A), and 22 genes were significantly down-regulated (S2C Fig) in ΔphoR -H37Rv as a function of acidic pH. Note that under identical conditions 167 genes showed upregulation whereas 89 genes were downregulated in WT-H37Rv. (TIF) [file pgen.1011070.s002.tif]

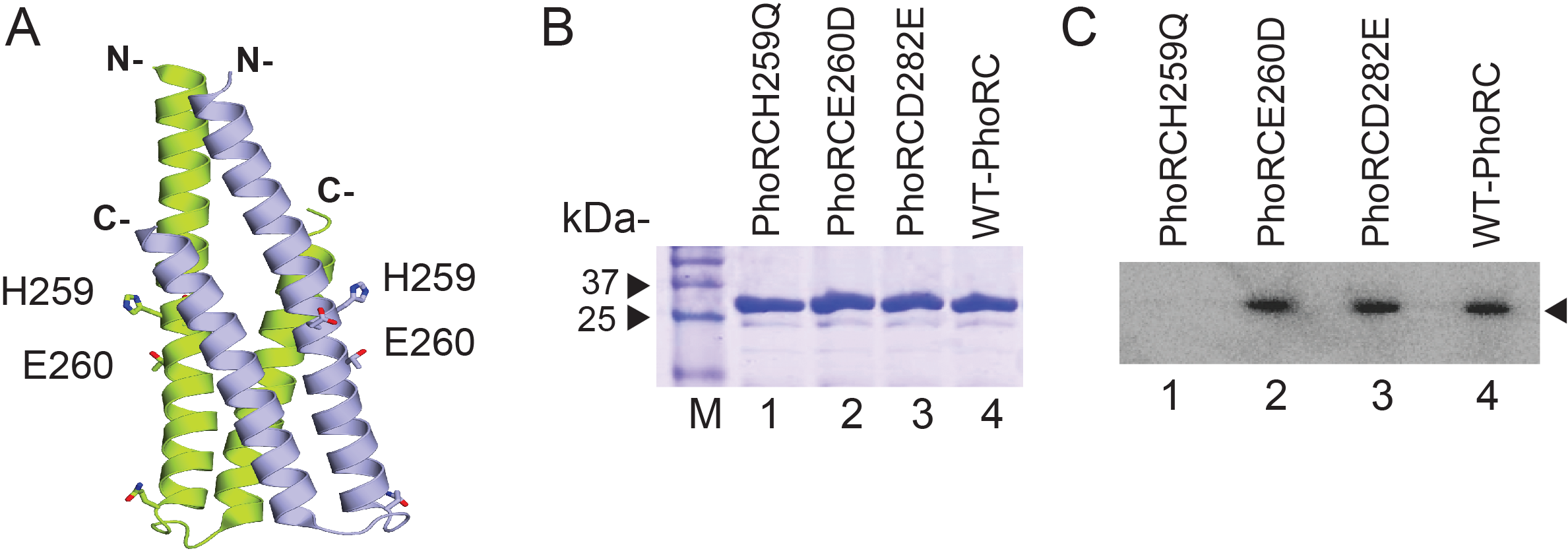

Supplement: S4 Fig — (A) The structural model of PhoRC DHp domain utilized structural coordinates of the SK [PDB ID: 5UKY] and was generated by Pymol (http://www.pymole.org/). The two most conserved residues are indicated on the figure. (B) Four recombinant PhoRC mutants, each carrying a conservative substitution of a single amino acid residue of PhoR DHp domain, were cloned, expressed and purified as described in the Materials and Methods. Purified proteins (≈2 μg/lane) were analyzed by SDS/polyacrylamide gel electrophoresis and visualized by Coommassie blue staining. The sizes of the molecular mass markers (in kDa) are indicated to the left of the figure. See ‘Results‘ section for a description of the PhoRC mutants. (C) The indicated recombinant mutants were purified and auto-phosphorylated as described in the methods. The products were resolved by SDS-PAGE and digitized by a phosphorimager. (TIF) [file pgen.1011070.s004.tif]

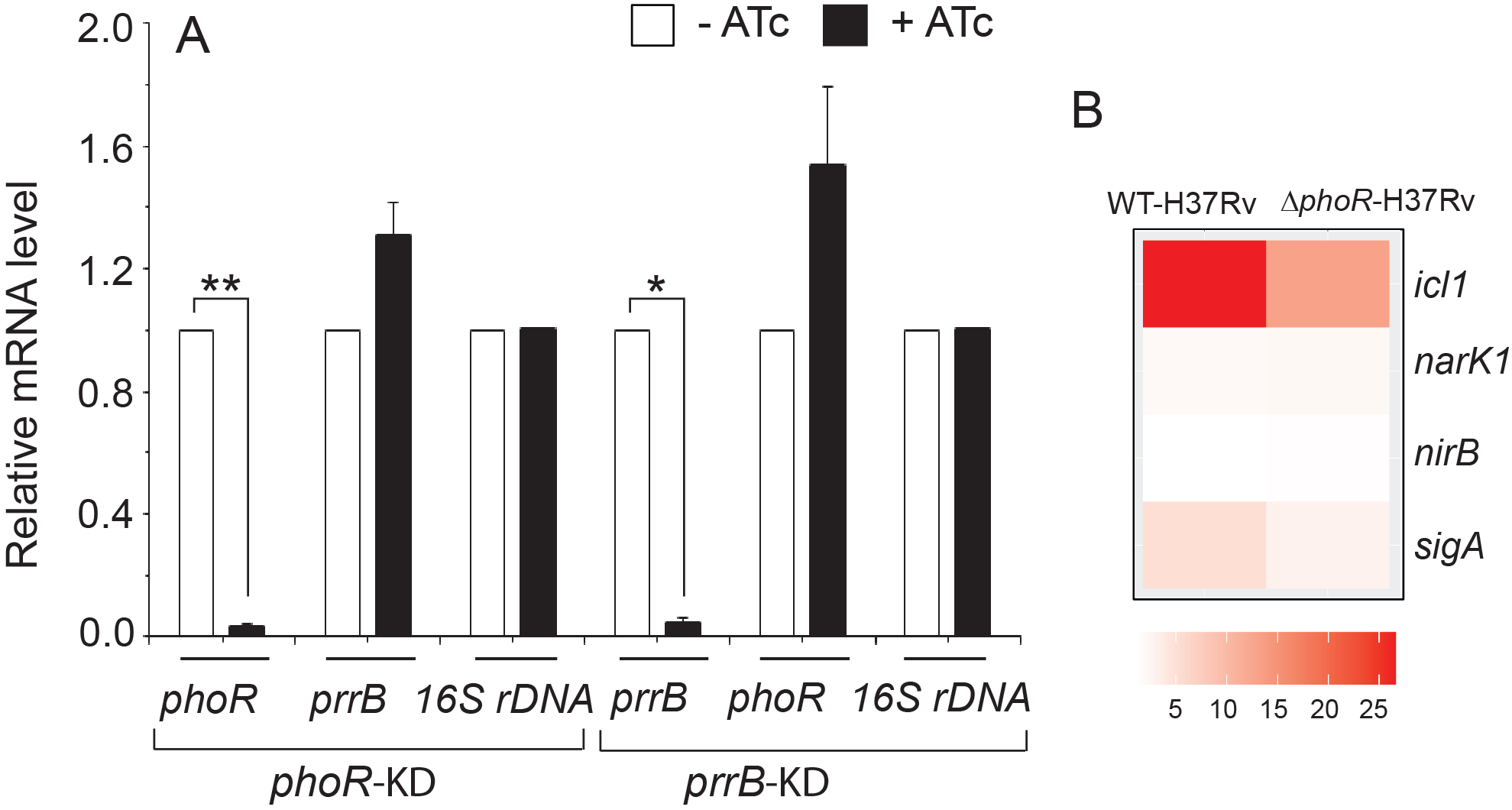

Supplement: S5 Fig — (A) Expression levels of indicated genes in phoR and prrB knock-down constructs grown under acidic pH (pH 4.5) conditions of growth. To compare respective mRNA levels, gene-specific expression was determined by RT-qPCR (see Methods). The results display average values derived from biological duplicates, each performed with two technical repeats (*P≤0.05; **P≤0.01; ***P≤0.001). (B) To compare relative expression of a few representative genes, RNA-seq data from WT-H37Rv and ΔphoR-H37Rv, grown under acidic pH conditions of growth, were analysed and shown as a heat-map. (TIF) [file pgen.1011070.s005.tif]
