# Supplementary material for: Dual functioning by the PhoR sensor is a key determinant to Mycobacterium tuberculosis virulence
Source: PLoS Genet. 2023 Dec 15;19(12):e1011070. doi: 10.1371/journal.pgen.1011070 (PMC10723718; doi:10.1371/journal.pgen.1011070)
Supplement: S1 Table — (DOCX) [file pgen.1011070.s006.docx]

**S1 Table**

Oligonucleotide primers used for cloning and amplifications reported in this study

| **Primers^a^** | **Sequence or description (5' - 3')** | **Reference** |
| --- | --- | --- |
| FPphoRLHR | TTTTTTTTCCATAAATTGGAGTACTGTGTCGGCGGCGGCGA | This study |
| RPphoRLHR | TTTTTTTTCCATTTCTTGGCAGGCGTACCCGTA | This study |
| FPphoRRHR | TTTTTTTTCACAGAGTGGACCAGCTGAGCCTCA | This study |
| RPphoRRHR | TTTTTTTTCACCTTGTGAGTACTCGAAGCTGTTGTCGATGT | This study |
| FPhrcAstart | AATAATCATATGATGGGAAGCGCCGA | [1] |
| RPhrcAstop | AATAATGGATCCTCATCGAGCACCCA | [1] |
| FPPrrA | ATATAGGATCCCACCACCACCACCACCACATGGGCGGCATGGACAC | This study |
| RPPrrA | AATAATAAGCTTTCACTGCATAC | This study |
| FPPrrB | ATATAGGATCCATGAATATTCTGTC | This study |
| RPPrrB | ATATAAAAGCTTTCACACCACCACCACCACCACACTGGGCCCGGGAAGGC | This study |
| FPphoRH259Q | ATCACCGACGCCAGCCAAGAACTGC | This study |
| RPphoRH259Q | GCAGTTCATGGCTGGCGTCGGTGAT | This study |
| FPphoRE260D | ACCGACGCCAGCCATGAGCTGCGTACCC | This study |
| RPphoRE260D | GGGTACGCAGCTCATGGCTGGCGTCGGT | This study |
| FPphoRD282E | CAAGGAGCCGCCCGCGAGGTGGG | This study |
| RPphoRD282E | CCCACCTCGCGGGCGGCTCCTTGT | This study |
| FPphoRC | GTTATACATATGACGGCCGCGGCGATC | [2] |
| RPphoRC | GGTGGTGGATCCTTATCAGGGCGGCCCTGGCACAAC | [2] |
| FPmphoP | GTTTGCGGATCCATGCGGAAAGGGGTTGAT | [3] |
| RPmphoP | GGTGGTCTGCAGTCAGTGGTGGTGGTGGTGGTGTCGAGGCTCCCGCAG | [3] |
| FPphoRcomp | aataatcatatggcgagccccgct | This study |
| RPphoRcomp | aataatctgcaggggcggccctggca | This study |
| FPphoRInt | CCCTACCCTGCGCATAACCCC | This study |
| RPphoRInt | ACGCAGTTCCGCGCTGGCGTC | This study |
| FPPrrBC | ATATATCATATGGCCAGCGAAGCCA | This study |
| RPprrBC | ATATATAAGCTTCTAACTGGGCCCG | This study |
| FPphoPD71N | GTGATCCTCAACGTGATGATGCCC | [2] |
| RPphoPD71N | CATCATCACGTTGAGGATCACCGC | [2] |
| FPdosR | AATAATCATATGATGGGAAGCGCCGA | [4] |
| RPdosR | AATAATGGATCCTCATCGAGCACCCA | [4] |
| 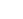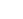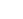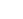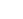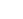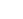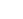FPphoRsg | 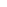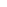GGGAAGTGGCCACCAGGATCAGCG | This Study |
| 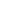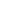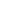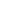RPphoRsg | AAACCGCTGATCCTGGTGGCCACT | This Study |
| FPprrBsg | GGGAGGACCGTGATGAGGGCGTCC | This Study |
| 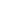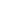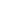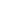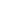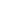RPprrBsg | AAACGGACGCCCTCATCACGGTCC | This Study |
| 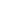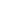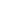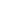FPpRH2521seq | 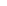AAACTCTAGAAATATTGGATCG | This study |
| 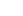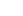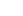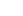RPpRH2521seq | CCTAATGACCATGGTGACCTC | This study |

^a^FP, forward primer; RP, reverse primer

**References**

1. Sevalkar RR, Arora D, Singh PR, Singh R, Nandicoori VK, Karthikeyan S, et al. Functioning of Mycobacterial Heat Shock Repressors Requires the Master Virulence Regulator PhoP. Journal of bacteriology. 2019;201(12). Epub 2019/04/10. doi: 10.1128/JB.00013-19. PubMed PMID: 30962357; PubMed Central PMCID: PMC6531620.

2. Gupta S, Sinha A, Sarkar D. Transcriptional autoregulation by Mycobacterium tuberculosis PhoP involves recognition of novel direct repeat sequences in the regulatory region of the promoter. FEBS letters. 2006;580(22):5328-38. Epub 2006/09/19. doi: 10.1016/j.febslet.2006.09.004. PubMed PMID: 16979633.

3. Anil Kumar V, Goyal R, Bansal R, Singh N, Sevalkar RR, Kumar A, et al. EspR-dependent ESAT-6 Protein Secretion of Mycobacterium tuberculosis Requires the Presence of Virulence Regulator PhoP. The Journal of biological chemistry. 2016;291(36):19018-30. Epub 2016/07/23. doi: 10.1074/jbc.M116.746289. PubMed PMID: 27445330; PubMed Central PMCID: PMC5009273.

4. Singh PR, Vijjamarri AK, Sarkar D. Metabolic Switching of Mycobacterium tuberculosis during Hypoxia Is Controlled by the Virulence Regulator PhoP. Journal of bacteriology. 2020;202(7). Epub 2020/01/15. doi: 10.1128/JB.00705-19. PubMed PMID: 31932312; PubMed Central PMCID: PMC7167471.
