# Supplementary material for: Dual functioning by the PhoR sensor is a key determinant to Mycobacterium tuberculosis virulence
Source: PLoS Genet. 2023 Dec 15;19(12):e1011070. doi: 10.1371/journal.pgen.1011070 (PMC10723718; doi:10.1371/journal.pgen.1011070)
Supplement: S2 Table — (DOCX) [file pgen.1011070.s007.docx]

**S2 Table**

Plasmids used for cloning and expression reported in this study

| **Plasmids^b^** |  |  |
| --- | --- | --- |
| pET15b | Plasmid used as a cloning vector in *E. coli*, Amp^r, a^ | Novagen |
| pET-*phoP* | pET15b expressing His_6_-tagged PhoP | [1] |
| pET-*phoPN* |  |  |
| pET-*phoPD71N* | Asp-71 codon mutated to Asn in pET-*phoP* | [1] |
| pET-28b | *E. coli* cloning vector, Kan^r, b^ | Novagen |
| pET-*phoRC* | pET-28b expressing His_6_-tagged PhoR (aa 193-485) | This study |
| pET-*dosR* | pET28b expressing His_6_-tagged DosR (aa 1-217) | [2] |
| pET-*prrBC* | pET28b expressing His_6_-tagged PrrB (aa 200-446) | This study |
| pET*-phoRCH259Q* | His-259 codon mutated to Gln in pET-PhoRC | [1] |
| pET*-phoRCE260D* | Glu-260 codon mutated to Asp in pET-PhoRC | This study |
| pET*-phoRCD282E* | Asp-282 codon mutated to Glu in pET-PhoRC | This study |
| p19Kpro | Mycobacterial expression vector, Hyg^r, c^ | [3] |
| p19Kpro-*phoP* | His_6_-tagged PhoP expression from mycobacterial expression vector p19Kpro | [4] |
| pST-Ki | Integrative mycobacterial expression vector, Kan^r, b^ | [5] |
| pST-*phoP* | His_6_- and FLAG-tagged *phoP* cloned in pSTKi | This study |
| pRH2502 | Integrative mycobacterial expression vector, Kan^r, b^ | [6] |
| pRH2521 | Episomal expression vector, Hyg^r, c^ | [6] |
| pRH2521-PhoRsg | pRH2521 vector expressing PhoR guide RNA, Hyg^r,c^ | This study |
| pRH2521-PrrBsg | pRH2521 vector expressing PrrB guide RNA, Hyg^r,c^ | This study |

Amp^r^, ampicillin resistance^a^; Kan^r^, kanamycin resistance^b^; Hyg^r^, hygromycin resistance^c^;

**References**

1. Gupta S, Sinha A, Sarkar D. Transcriptional autoregulation by Mycobacterium tuberculosis PhoP involves recognition of novel direct repeat sequences in the regulatory region of the promoter. FEBS letters. 2006;580(22):5328-38. Epub 2006/09/19. doi: 10.1016/j.febslet.2006.09.004. PubMed PMID: 16979633.

2. Singh PR, Vijjamarri AK, Sarkar D. Metabolic Switching of Mycobacterium tuberculosis during Hypoxia Is Controlled by the Virulence Regulator PhoP. J Bacteriol. 2020;202(7). Epub 2020/01/15. doi: 10.1128/jb.00705-19. PubMed PMID: 31932312; PubMed Central PMCID: PMCPMC7167471.

3. De Smet KA, Kempsell KE, Gallagher A, Duncan K, Young DB. Alteration of a single amino acid residue reverses fosfomycin resistance of recombinant MurA from Mycobacterium tuberculosis. Microbiology. 1999;145 ( Pt 11):3177-84. Epub 1999/12/10. doi: 10.1099/00221287-145-11-3177. PubMed PMID: 10589726.

4. Anil Kumar V, Goyal R, Bansal R, Singh N, Sevalkar RR, Kumar A, et al. EspR-dependent ESAT-6 Protein Secretion of Mycobacterium tuberculosis Requires the Presence of Virulence Regulator PhoP. The Journal of biological chemistry. 2016;291(36):19018-30. Epub 2016/07/23. doi: 10.1074/jbc.M116.746289. PubMed PMID: 27445330; PubMed Central PMCID: PMC5009273.

5. Parikh A, Kumar D, Chawla Y, Kurthkoti K, Khan S, Varshney U, et al. Development of a new generation of vectors for gene expression, gene replacement, and protein-protein interaction studies in mycobacteria. Applied and environmental microbiology. 2013;79(5):1718-29. Epub 2013/01/15. doi: 10.1128/AEM.03695-12. PubMed PMID: 23315736; PubMed Central PMCID: PMC3591980.

6. Singh AK, Carette X, Potluri LP, Sharp JD, Xu R, Prisic S, et al. Investigating essential gene function in Mycobacterium tuberculosis using an efficient CRISPR interference system. Nucleic Acids Res. 2016;44(18):e143. Epub 2016/07/14. doi: 10.1093/nar/gkw625. PubMed PMID: 27407107; PubMed Central PMCID: PMCPMC5062980.
