# Supplementary material for: Dual functioning by the PhoR sensor is a key determinant to Mycobacterium tuberculosis virulence
Source: PLoS Genet. 2023 Dec 15;19(12):e1011070. doi: 10.1371/journal.pgen.1011070 (PMC10723718; doi:10.1371/journal.pgen.1011070)
Supplement: S5 Table — (DOCX) [file pgen.1011070.s010.docx]

**S5 Table**

Sequences of oligonucleotides utilized in RT-PCR and ChIP results reported in this study

| **Primers^a^** | **Sequence or description (5’-3’)** | **Reference** |
| --- | --- | --- |
| FPaprART | TTGACCATGACAGCGAGTGT | This study |
| RPaprART | TTGGACAGAAATGCAGGATG | This study |
| FPethRRT | CCGGGATCAACGTGTTCTTC | This study |
| RPethRRT | CGCTCGTTCATCAGGTTGAG | This study |
| FPicl1RT | GCTTCTACCGCACCAAGAAC | [1] |
| RPicl1RT | TCGAGGTGCTTTTTCCAGTT | [1] |
| FPnarK1RT | ATCGTGTTGCTAATTCCGGC | [2] |
| RPnarK1RT | TTGCCAACAACCCAACTAGC | [2] |
| FPnirBRT | GTCAGCGGATCGACTTGTTC | [2] |
| RPnirBRT | GCTTTATTTTGTGCGGTGCC | [2] |
| FPphoRRT | GCCAGAGCTATACGGCACTC | This study |
| RPphoRRT | ACGGTGGTCAAGTAGCCATC | This study |
| FPpks2RT | GTTGTGGAAGGCGTTGTTAC | [3] |
| RPpks2RT | GTCGTAGAACTCGTCGCAAT | [3] |
| FPmsl3RT | GTGAAAACAAACTTCGGTCAC | [3] |
| RPmsl3RT | ACAAAGAGTTCAGTGTCAATCTCAG | [3] |
| FPRv0068RT | GACCGGCCTGTTGATTGATC | This study |
| RPRv0068RT | GCTAACCGACGCTGAAGTTC | This study |
| FPRv1217RT | CGTGCTGGCCTATCGATTAC | This study |
| RPRv1217RT | GTGTTATCGCCCAGCTGATC | This study |
| FPRv1219RT | CGAAAACCCAACCGATCTCC | This study |
| RPRv1219RT | CCGACATGTGCTTCTCCTTG | This study |
| FPRv2660RT | CAGCAACAGGCCAGGCTA | This study |
| RPRv2660RT | CTAGTGAAACTGGTTCAATCCCA | This study |
| FPRv3269RT | CTTCGTCAGACCGCGGTAT | This study |
| RPRv3269RT | CTGATCGCTGGAGTGGGC | This study |
| FPgapdhRT | AGTAGGCATCAACGGGTTTG | [4] |
| RPgapdhRT | GTGCTGTTGTCGGTGATGTC | [4] |
| FPsigART | ACTTTGCTGCAGGATCAACT | [5] |
| RPsigART | AACTTCGACATAGTCTTGGATTC | [5] |
| FP16SrDNA RT | CTGAGATACGGCCCCCAGACTC | [6] |
| RP16SrDNA RT | CGTCGATGGTGAAAGAGGTT | [6] |
| FPicl1up | AATAATAAGCTTACCGGATCCGCA | [1] |
| RPicl1up | AATAATGGTACCGTTCGTGTCC | [1] |
| FPmsl3up | GGTGGTGGATCCTGCATGCTGCTGTGG | [3] |
| RPmsl3up | GTTGTTGGTACCCAGCACGACGAAGAA | [3] |
| FPnarK1up | GTACTCGAGCACAATAGCTTTC | [2] |
| RPnarK1up | CGAAGGGGCCGCGGGACTGC | [2] |
| FPpe20up | GGCGAAGAAAGTAACCGTCA | This study |
| RPpe20up | CACCCATTGCTTCAGGTCG | This study |
| FPRv1219up | ATCCTACGACCTGGTATTCA | This study |
| RPRv1219up | TCGCCCAGCGGCACCAGTG | This study |
| FPRv2390up | TTTCACGATGATCAAAACTACGC | This study |
| RPRv2390up | CGGTGCCGCCTGGCTCACT | This study |
| FPwhiB3up | TGGACTCATCGATGTTCTTCC | This study |
| RPwhiB3up | TAGGGCTCACCGACCTCTAA | This study |
| FPRpoBup | GGAGGCGATCACACCGCAGACGT | This study |
| RPRpoBup | CCTCCAGCCCGGCACGCTCACGT | This study |
| FP16SrDNAup | CTGAGATACGGCCCAGAGCTC | [2] |
| RP16SrDNAup | CTCCGATGGTGAAAGAGGTT | [2] |

^a^FP: forward primer; RP: reverse primer

**References**

1. Khan H, Paul P, Sevalkar RR, Kachhap S, Singh B, Sarkar D. Convergence of two global regulators to coordinate expression of essential virulence determinants of Mycobacterium tuberculosis. Elife. 2022;11. Epub 2022/11/10. doi: 10.7554/eLife.80965. PubMed PMID: 36350294; PubMed Central PMCID: PMCPMC9645806.

2. Singh PR, Vijjamarri AK, Sarkar D. Metabolic Switching of Mycobacterium tuberculosis during Hypoxia Is Controlled by the Virulence Regulator PhoP. Journal of bacteriology. 2020;202(7). Epub 2020/01/15. doi: 10.1128/JB.00705-19. PubMed PMID: 31932312; PubMed Central PMCID: PMC7167471.

3. Goyal R, Das AK, Singh R, Singh PK, Korpole S, Sarkar D. Phosphorylation of PhoP protein plays direct regulatory role in lipid biosynthesis of Mycobacterium tuberculosis. J Biol Chem. 2011;286(52):45197-208. Epub 2011/11/11. doi: 10.1074/jbc.M111.307447. PubMed PMID: 22072719; PubMed Central PMCID: PMC3247998.

4. Gupta S, Sinha A, Sarkar D. Transcriptional autoregulation by Mycobacterium tuberculosis PhoP involves recognition of novel direct repeat sequences in the regulatory region of the promoter. FEBS letters. 2006;580(22):5328-38. Epub 2006/09/19. doi: 10.1016/j.febslet.2006.09.004. PubMed PMID: 16979633.

5. Bansal R, Anil Kumar V, Sevalkar RR, Singh PR, Sarkar D. Mycobacterium tuberculosis virulence-regulator PhoP interacts with alternative sigma factor SigE during acid-stress response. Molecular microbiology. 2017;104(3):400-11. Epub 2017/02/01. doi: 10.1111/mmi.13635. PubMed PMID: 28142206.

6. Singh R, Anil Kumar V, Das AK, Bansal R, Sarkar D. A transcriptional co-repressor regulatory circuit controlling the heat-shock response of Mycobacterium tuberculosis. Mol Microbiol. 2014;94(2):450-65. Epub 2014/08/30. doi: 10.1111/mmi.12778. PubMed PMID: 25171378.
