# Supplementary material for: Dual functioning by the PhoR sensor is a key determinant to Mycobacterium tuberculosis virulence
Source: PLoS Genet. 2023 Dec 15;19(12):e1011070. doi: 10.1371/journal.pgen.1011070 (PMC10723718; doi:10.1371/journal.pgen.1011070)
Supplement: S6 Table — (DOCX) [file pgen.1011070.s011.docx]

**S6 Table**

Sequences of oligonucleotides used in M-PFC experiments reported in this study

| **^a^Primers** | **Sequence or description (5’-3’)** | **Reference** |
| --- | --- | --- |
| FPphoP | AATAATCAATTGATGCGGAAAGGG | [1] |
| RPphoP | AATAATAAGCTTTCATCGAGGCTC | [1] |
| FPphoR | AATAAACTGCAGATGGCCAGACAC | [1] |
| RPphoR | AATAATAAGCTTTCAGGGCGGCC | [1] |
| FPdosS | AATAATGGATCCATGACAACAGGGG | This study |
| RPdosS | AATAATAAGCTTCTACTGCGACAAC | This study |
| FPdosT | AATAATGGATCCGTGACACACCCTG | This study |
| RPdosT | AATAATAAGCTTTCAGCGCAGCGGT | This study |
| FPkdpD | AATAATGGATCCGTGACGTTGCTCT | This study |
| RPkdpD | AATAATAAGCTTTCATGGGCGGTCC | This study |
| FPmprB | AATAATGGATCCATGTGGTGGTTCC | This study |
| RPmprB | AATAATAAGCTTCTAGGTTGCGCGC | This study |
| FPmtrB | AATAATAAGCTTATGATCTTCGGCT | This study |
| RPmtrB | AATAATATCGATTCAACCGCTCCAC | This study |
| FPpdtaS | AATAATGGATCCATGTCCACACTCG | This study |
| RPpdtaS | AATAATAAGCTTCTACAGCATCAAC | This study |
| FPprrB | AATAATGGATCCATGAATATTCTGT | This study |
| RPprrB | AATAATAAGCTTCTAACTGGGCCCG | This study |
| FPsenX3 | AATAATGGATCCGTGACTGTGTTC | This study |
| RPsenX3 | AATAATAAGCTTTCATCGGCTCAGC | This study |
| FPtcrY | AATAATGGATCCGTGGGAATCACCG | This study |
| RPtcrY | AATAATAAGCTTCTAGCGCGGCGAC | This study |
| FPtrcS | AATAATGGATCCATGATCCCGGACC | This study |
| RPtrcS | AATAATAAGCTTTCAGGCGGTAGTG | This study |
| FPRv0060c | AATAATGGATCCGTGCCGATCACTC | This study |
| RPRv0060c | AATAATAAGCTTCTATTGGTCGCTA | This study |
| FPRv0601c | AATAATCTGCAGATGGCGCTCGTTC | This study |
| RPRv0601c | AATAATAAGCTTTCAGCTGGTCGGC | This study |
| FP0845c | AATAATGGATCCGTGCCCAGCTACG | This study |
| RP0845c | AATAATAAGCTTTCACCGTTTCAGT | This study |
| FPRv3220c | AATAATGGATCCATGTCCACACTCG | This study |
| RPRv3220c | AATAATAAGCTTCTACAGCATCCAC | This study |

^a^FP: forward primer; RP: reverse primer

**References**

1. Bansal R, Anil Kumar V, Sevalkar RR, Singh PR, Sarkar D. Mycobacterium tuberculosis virulence-regulator PhoP interacts with alternative sigma factor SigE during acid-stress response. Molecular microbiology. 2017;104(3):400-11. Epub 2017/02/01. doi: 10.1111/mmi.13635. PubMed PMID: 28142206.
