# Supplementary material for: Dual functioning by the PhoR sensor is a key determinant to Mycobacterium tuberculosis virulence
Source: PLoS Genet. 2023 Dec 15;19(12):e1011070. doi: 10.1371/journal.pgen.1011070 (PMC10723718; doi:10.1371/journal.pgen.1011070)
Supplement: S7 Table — (DOCX) [file pgen.1011070.s012.docx]

**S7 Table**

Plasmids used in M-PFC experiments reported in this study

| **Plasmids** | **Relevant details** | **References** |
| --- | --- | --- |
| pUAB400^a^ | Integrative expression plasmid, Kan^r^ | [1] |
| pUAB-*phoP* | pUAB400 expressing PhoP (aa 1-247) | [2] |
| pUAB300^b^ | Episomal expression plasmid, Hyg^r^ | [1] |
| pUAB300-*phoR* | pUAB300 expressing PhoR (aa 1-485) | [2] |
| pUAB300-*dosS* | pUAB300 expressing DosS (aa 1-579) | This study |
| pUAB300-*dosT* | pUAB300 expressing DosT (aa 1-574) | This study |
| pUAB300-*kdpD* | pUAB300 expressing KdpD (aa 1-861) | This study |
| pUAB300-*mprB* | pUAB300 expressing MprB (aa 1-505) | This study |
| pUAB300-*mtrB* | pUAB300 expressing MtrB (aa 1-568) | This study |
| pUAB300-*pdtaS* | pUAB300 expressing PdtaS (aa 1-502) | This study |
| pUAB300-*prrB* | pUAB300 expressing PrrB (aa 1-447) | This study |
| pUAB300-*senX3* | pUAB300 expressing SenX3 (aa 1-411) | This study |
| pUAB300-*tcrY* | pUAB300 expressing TcrY (aa 1-476) | This study |
| pUAB300-*trcS* | pUAB300 expressing TrcS (aa 1-510) | This study |
| pUAB300-*Rv0600c* | pUAB300 expressing Rv0600c (aa 1-169) | This study |
| pUAB300-*Rv0601c* | pUAB300 expressing Rv0601c (aa 1-157) | This study |
| pUAB300-*Rv0845c* | pUAB300 expressing Rv0845c (aa 1-426) | This study |
| pUAB300-*Rv3220c* | pUAB300 expressing Rv3220c (aa 1-502) | This study |

^a^ kanamyin resistance; ^b^ hygromycin resistance

**References**

1. Singh A, Mai D, Kumar A, Steyn AJ. Dissecting virulence pathways of Mycobacterium tuberculosis through protein-protein association. Proc Natl Acad Sci U S A. 2006;103(30):11346-51. Epub 2006/07/18. doi: 10.1073/pnas.0602817103. PubMed PMID: 16844784; PubMed Central PMCID: PMC1544089.

2. Singh R, Anil Kumar V, Das AK, Bansal R, Sarkar D. A transcriptional co-repressor regulatory circuit controlling the heat-shock response of Mycobacterium tuberculosis. Mol Microbiol. 2014;94(2):450-65. Epub 2014/08/30. doi: 10.1111/mmi.12778. PubMed PMID: 25171378.
