# Supplementary material for: Dual functioning by the PhoR sensor is a key determinant to Mycobacterium tuberculosis virulence
Source: PLoS Genet. 2023 Dec 15;19(12):e1011070. doi: 10.1371/journal.pgen.1011070 (PMC10723718; doi:10.1371/journal.pgen.1011070)
Supplement: Attachment — Submitted filename: Response to Reviewers Oct 2023.docx [file pgen.1011070.s013.docx]

**Reviewer #1:**

**General comment:**

*Reviewer #1: The manuscript “Dual functioning by the PhoR sensor is a key determinant to Mycobacterium tuberculosis virulence” by Singh et al, revisits a very important question in the mycobacterial TCS crosstalk that have from time to time shown to play a very important role in adaptation and survival of the bacteria to various stresses. In this work the authors have shown that the PrrB can act as a non-canonical phosphate donor*

*PhoP. They have also explored the dual function (kinase and phosphatase) of the PhoR. First, I would like to say that the paper is very well written, especially mentioning the lanes in text is highly appreciable as it makes it easy for the readers to understand. The labelling of the figures is also very good. My only suggestion would be to include the non-cognate PrrB axis later in the paper just before the macrophage and mice experiment. This will help the readers to keep up with the flow. Despite some interesting observations, given below are some of my comments and questions that must be addressed before publication.*

We thank this reviewer for the nice and extremely positive comments.

We find reviewer’s suggestion to include the non-cognate PrrB axis later in the manuscript, an interesting one. We have now rearranged the flow, and the figures and results have been adjusted accordingly in the revised manuscript.

**Major comments***:*

*#1: It is already known that PhoP is instrumental in acid stress (Bansal et al 2017) and PhoR is the cognate partner of PhoP. From Fig 1 the takeaway message is that PhoR is getting phosphorylated at acidic condition and in absence of PhoR there is no *P-PhoP seen. Yes, previous studies have either worked on ΔphoP or ΔphoPR system leaving a confusion of the role of PhoR. MPFC and pull-down assay shows interaction of PrrB with PhoP. Then in Fig 4 it is shown that PrrB can phosphorylate PhoP. So my question is why is there no detectable *P-PhoP in ΔphoR::phoP (Fig 2C)?*

We would like to clarify that although PhoP is phosphorylated by PrrB (Fig. 6B) and PrrB is partially phosphorylated under acidic pH (Fig. 6E), we were unable to conclusively demonstrate that PrrB phosphorylates PhoP in response to acidic conditions. In fact, we do not think PrrB functions as a sensor of acidic pH, rather it is partly phosphorylated in response to a signal that mimics altered physiology under acidic pH. Note that in the mycobacterial cell lysates while 100% of the PhoR protein was present in the phosphorylated form, only ~35% PrrB gets phosphorylated under identical acidic conditions of growth (Fig. 6E). Perhaps that is why even under acidic pH, PhoP remains unphosphorylated in a mutant strain lacking *phoR*.

This has now been explained in more detail in the ‘Discussion’ section (lines 511-525) of the revised manuscript (duplicated here):

“Note that the environmental signal(s) that triggers specific and non-canonical mode of PhoP activation via PrrB, requiring an appropriate transcriptional response, remains unknown. Although we show that PhoP is phosphorylated by PrrB (Fig. 6B) and PrrB is partially phosphorylated under acidic pH (Fig. 6E), we were unable to conclusively demonstrate that PrrB phosphorylates PhoP in response to acidic conditions. In fact, we do not think PrrB functions as a sensor of acidic pH; rather we speculate that PrrB is partly phosphorylated in response to a signal that mimics altered physiology under acidic pH, for the following reasons. First, recombinant PrrBC in a robust reaction can phosphorylate PhoPN *in vitro* (Fig. 6B). Second, in the mycobacterial cell lysates while 100% of the PhoR protein was present in the phosphorylated form, only ~35% PrrB gets phosphorylated *in vivo* under identical acidic conditions of growth (Fig. 6E). Third, and most importantly, a set of acidic pH -inducible PhoP regulon genes are significantly down-regulated in a *prrB*-KD mutant, but not in a *phoR*-KD strain (Figs. 6C-D). Together, these results, for the first time, connect two SKs, PhoR and PrrB, to a single RR (PhoP) in a stress-specific manner and provide new biological insights into mechanism of activation of the versatile regulator.”

*#2: Going by the RNA seq data, the gene list shown in Fig 3A, is it specific for ΔphoR only? What is the number of overlapping genes between ΔphoP and ΔphoR? Also, what number of genes were down regulated in ΔphoR under acidic condition? Authors should also include a heat map of the downregulated genes as shown in fig 3A. Moreover, a Venn diagram showing the list of genes in ΔphoP and ΔphoR along with overlapping gene sets should also be include be better understanding on the control of PhoP and PhoR. This should be followed by a proper discussion.*

Fig. 3A represents a heat map showing over-expression of acid inducible genes under low pH conditions relative to normal conditions (pH 7.0) of growth. As we compared significantly over-expressed genes under low pH in *ΔphoR* mutant, most of these genes are likely to be specific for the *ΔphoR* mutant only.

We have now included a Venn diagram displaying number of genes independently regulated by PhoR and PhoP (Fig. S2C). Further, a heat-map showing a list of down-regulated genes under acidic pH conditions in *ΔphoR* mutant was included as a new supplemental figure (Fig. S2D). While transcriptomic data of *phoP* mutant was taken from Solans et al., 2014, the RNA sequencing data of *phoR* mutant from this study was used for comparative analysis. Finally, new text was included to discuss these results in the revised manuscript.

Reference:

Solans L, Gonzalo-Asensio J, Sala C, Benjak A, Uplekar S, Rougemont J, Guilhot C, Malaga W, Martin C, Cole ST. (2014) The PhoP-dependent ncRNA Mcr7 modulates the TAT secretion system in Mycobacterium tuberculosis. PLoS Pathog 10:e1004183.

*#3: Line 267-268, the authors have mentioned that for their in vitro phosphotransfer assay, they have specifically used the N terminal of PhoP and C terminal of PrrB. What was the reason for the authors to select such domain specific region? Will there be any change in results if full length proteins were to be used? In fact, using full length proteins is highly feasible due to significant molecular weight difference between PrrB and PhoP (PrrB is 47.8kd and PhoP is 27.5 kd). If there is any ref paper that has characterized the ATP binding pockets of PrrB that is being followed, then the authors should specify that. Fig 5B should include a control between *P-PhoRC and PhoPN.*

The reviewer seeks justification of use of domain constructs of the RR and SK proteins instead of their full-length counterparts during *in vitro* phosphotransfer assays (Fig. 6B).

This has now been explained in more detail in the ‘Results’ section (duplicated here) (lines 335-340):

“To facilitate purification, full-length PhoP and the cytoplasmic portion of PhoR (PhoRC) was over-expressed as His_6_-tagged fusion proteins, as described previously (Gupta et al., 2006). An N-terminally truncated form of PhoR (PhoRC, residues 193 to 485) and PrrB (PrrBC, residues 200 to 446) lacking the membrane spanning region were chosen, as over-expression of full-length sensor kinase genes is often toxic (Haydel et al., 1999; Himpens et al., 2000). Additionally, PhoRC, and PrrBC were preferred to avoid insolubility due to the presence of hydrophobic amino acid stretches.”

PrrBC was constructed based on a sequence alignment result with members of the SK family of proteins.

Note that full length PhoP (247 amino acids) is comparable to the cytoplasmic region of PrrB protein (PrrBC comprising 247 amino acids) in size, justifying use of the N-terminal domain of PhoP (PhoPN, 141 amino acids), which was previously shown to function as the phosphoacceptor domain as effectively as the full-length PhoP (Pathak et al., 2010).

As requested, we have now included a control data set showing phosphotransfer between labelled PhoRC and PhoPN (Fig. 6B, revised manuscript).

References:

Gupta, S., Sinha, A., and Sarkar, D. (2006) Transcriptional autoregulation of *Mycobacterium*

*tuberculosis* PhoP involves recognition of novel direct repeat sequences in the regulatory

region of the promoter. FEBS Lett 580, 5328-5338

Haydel, S.E., Dunlap, N.E. and W.H. Benjamin, Jr. (1999) *In vitro* evidence of two-component system phosphorylation between the *Mycobacterium tuberculosis* TrcR/TrcS proteins. Microb. Pathog. 26, 195-206

Himpens, S., Locht, C. and Supply, P. (2000) Molecular characterization of the

mycobacterial SenX3-RegX3 two-component system: evidence for autoregulation.

Microbiology 146, 3091-3098

Pathak, A., Goyal, R., Sinha, A., and Sarkar, D. (2010) Domain structure of virulence-associated response regulator PhoP of *Mycobacterium tuberculosis*: role of the linker region on regulator-promoter interaction(s). J Biol Chem 285, 34309-34318

*#4: Fig 5C and 5D really make things confusing. Going by the observation it seems as if PhoR does not control PhoP regulon at acidic stress. This is a shift from the known literature. I would request the authors to reverify this observation once again.*

We apologize for the confusion. To address the reviewer’s concern in greater detail and to further support the significance of our work, we have now included additional data on expression profile of representative genes showing both *phoR*-dependent and *phoR*-independent robust pH-inducible expression. The figures from the previous version (Figs. 5C-D) are now replaced with new figures (Figs. 6C-D), and the relevant results have been adjusted accordingly in the revised manuscript.

This has now been explained in more detail in the ‘Results’ section (lines 357-379, revised manuscript) (duplicated here):

“Next, to further probe activation of PhoP by specific SKs, we adopted a CRISPRi- based gene knock-down approach (Singh et al., 2016). The *phoR* and *prrB* knock-down constructs are described in the Methods, and relative mRNA levels compared expression of genes of interest in the corresponding knock-down strains relative to WT-H37Rv (Fig. S5A). Note that both the *phoR*-KD and *prrB*-KD mutants displayed significantly down-regulated expression of the corresponding target genes relative to WT bacilli. However, as controls *prrB* expression in *phoR*-KD and *phoR* expression in *prrB*-KD showed insignificantly higher expression relative to the WT-bacteria. Next, we grew these strains under low pH conditions to examine expression of a few representative genes, which showed either *phoR* -dependent or *phoR*-independent pH-inducible activation (Tables S3-S4). Importantly, representative genes displaying *phoR*-dependent pH-inducible expression (see Fig. 3B) clearly showed a significantly lowered expression in *phoR*-KD strain relative to WT-H37Rv under acidic pH (Fig. 6C). However, consistent with the results reported in RNA-seq data (Fig. 3A), *phoR*-KD mutant failed to impact expression of the genes which show *phoR*-independent pH- inducible expression. Fig. S5B shows RNA-seq derived heat-map of these genes from WT- and *ΔphoR*-H37Rv, under low pH conditions of growth. More importantly, genes which remained unaffected in *phoR*-KD strain, showed a significant inhibition of expression in *prrB*-KD strain relative to the WT bacilli under identical conditions (Fig. 6D). In contrast, expression of genes showing significant inhibition in *phoR*-KD mutant, remained unaffected in *prrB*-KD, under identical conditions examined. Collectively, these data suggest that although PhoR accounts for activation of PhoP regulon under acidic pH, PhoP activation is partly attributable to mycobacterial PrrB. These results are consistent with PrrB -dependent phosphorylation of PhoP, and for the first time, connect two SKs to integrate signal-dependent activation of PhoP, perhaps uncovering an effective means of mycobacterial adaptability to varying intracellular environments.”

Reference:

Singh et al., (2016) Investigating essential gene function in Mycobacterium tuberculosis using an efficient CRISPR interference system. Nucleic Acids Res. 44:e143

*#5: Fig7A: please include a better resolution image of the confocal. No differences between the wT-H37Rv and phopR-H37Rv can be concluded from this representative image.*

We have now included a higher resolution confocal image to replace the original figure (please see Fig. 7A of the revised manuscript).

Minor comments:

*#1: Line 389: please make sure this is 330-fold, the graph looks different.*

The text including the above line is no longer part of the revised manuscript.

**Reviewer #2:**

**Major comments:**

*#1: Given the authors' pursuit of other kinases that may phosphorylate PhoP based on the continued presence of 35 genes that still exhibit induction upon acidic pH exposure in a ∆phoR mutant (Fig. 3A), it would be helpful if some of those 35 genes are included in the qRT-PCR and ChIP-qPCR experiments shown in Figs. 3B and 3C, to show complementation. This in particular as it appears from Table S3 that there is a range of behavior of these 35 genes in WT Mtb (i.e.it is not that the ∆phoR mutant behaved like WT in expression of these genes; rather, some of these genes were not induced in WT, some were induced more, and some were induced less).*

We have now included new additional data showing low pH-inducible expression of representative genes (from Fig. 3A) in the revised manuscript. Our results highlight that consistent with the RNA seq data, expression of these genes display *phoR* independent acidic pH-inducible expression (see Fig. S3A). Further, in keeping with the expression profile, ChIP-qPCR experiments demonstrate *phoR*-independent acidic pH-inducible PhoP recruitment within target promoters in *ΔphoR*-H37Rv (see Fig. S3B).

The new supplemental figures, figure captions and the relevant results have been adjusted accordingly in the revised manuscript.

*#2: With the M-PFC assay, are the 3 spots down in each panel supposed to represent triplicate spotting of the same sample? If so, can the authors explain why the results do not replicate, even in the case of the positive control (PhoP-PhoR interaction, lane 4, which also shows very weak positive signal, given the low TRIM concentration used)? Only one spot shows positive signal for the PhoP-PrrB test on the TRIM-containing plates, and the assay results as shown thus do not appear robust. Did the authors try tagging PhoP and/or the various histidine kinases on the opposite terminus to determine which pairings provide the most robust data (i.e. use of the pUAB100 and pUAB200 plasmids of the MPFC assay system)? The weak signal observed even for the positive control raises questions that other interactions might have been missed in this assay. It is appreciated in the case of the reported interaction between PhoP and PrrB that the co-immunoprecipitation results shown in Fig. 4C do however provide much stronger support.*

We apologize for the lack of clarity to the legend of Fig. 5A-B and have now provided additional explanation. The three spots moving downwards in each panel represent spotting of bacterial cells at three different dilutions, namely undiluted, 10-fold and 100-fold dilutions, respectively.

Although we performed these assays as independent replicates, we did not attempt interaction assays by expressing PhoP and sensor kinases from the other partners of the plasmid pair. Therefore, we have now explained it by including new text in the results section (lines 313-316) of the revised manuscript (duplicated here):

“Although we cannot rule out the possibility of other kinases with weaker interaction affinities for PhoP remaining undetectable in this assay, collectively these results, under the conditions examined, suggest specific protein-protein interaction between PrrB and PhoP.”

*#3: prrB knockdown also resulted in increased expression of phoR (Fig. S3A) – given this phenotype and the authors' finding that PhoR has phosphatase activity against phosphorylated PhoP, can the authors ascribe the effects shown in Fig. 5D solely to presumed phosphorylation of PhoP by PrrB? What is effect of prrB knockdown on phoR-dependent genes (genes shown in the qRT-PCR data in Fig. 3B)? The 3 genes selected for analysis in Figs. 5C-5D are all very weakly induced in acidic pH in the ∆phoR mutant. Was there a reason the authors did not examine genes that were more robustly induced in the ∆phoR mutant (e.g. from Fig. 3A: PE20, mvmT, cysK2 etc)?*

We understand the reviewer’s concerns related to results reported in Figs. 5C-D (Figs. 6C-D pf the revised version). Note that the increase in *phoR* expression level in *prrB* knock-down mutant and *prrB* expression level in *phoR* knock-down mutant, respectively remain insignificant (see Fig. S5A). We have now clearly mentioned it in the results section (lines 362-363).

Also, we have now included following changes in the revised manuscript.

(i) We have incorporated new data on expression profile of representative genes showing both *phoR*-dependent and *phoR*-independent low pH-inducible expression. The figures from the previous version (Figs. 5C-D) are now replaced with new figures (Figs. 6C-D), and the relevant results have been adjusted accordingly in the revised manuscript.

(ii) With regard to the reviewer’s query on whether the effects shown in Fig. 5D (of the original version) is attributable to phosphorylation of PhoP by PrrB, we have now included new text in the revised manuscript.

(a) Text included in the ‘Results’ section (lines 357-379, revised manuscript)

Please see our response to comment #4 of Reviewer 1.

(b) Text included in the ‘Discussion’ section (lines 511-525, revised manuscript)

Please see our response to comment #1 of Reviewer 1.

*#4: Along these lines, the conclusion in lines 312-313 is not explicitly supported by the current data – the authors have shown that PrrB can interact with PhoP and phosphorylate it in vitro, and PrrB is phosphorylated in acidic pH conditions in an intact bacterium, but whether PrrB is indeed then phosphorylating PhoP in the context of an intact wild type bacterium when exposed to acidic pH has not been established. Have the authors tried phos-tag gel assays examining PhoP phosphorylation status in intact WT, phoR knockdown, and prrB knockdown Mtb exposed to acidic pH? In the absence of such direct data, the conclusions made should at minimum be moderated.*

We agree and have now toned down our conclusion. We acknowledge our experiments do not provide direct proof that PrrB senses acidic pH and in turn, phosphorylates PhoP. Thus, we have included new text (lines 511-525) in the ‘Discussion’ section to replace original text (lines 312-313, original version).

Please see our response above to comment #1 of Reviewer 1.

Please note that we were unable to perform phos-tag gel assays using knock-down mutants since these strains already carrying two antibiotic resistance markers further required expression of affinity tagged PhoP protein.

**Minor comments:**

*#1: A ∆phoR mutant Mtb strain has already previously been shown to be attenuated for in vivo colonization in a murine infection model, as indeed the authors briefly reference (current reference 33). This should be better acknowledged however – for example, given this known phenotype, it would seem it is not just the reduced ability of the ∆phoR mutant Mtb to grow in macrophages that would lead one to perform the in vivo infection experiments (lines 385-386).*

We perfectly agree with the reviewer. We found it difficult to reconcile both the matters, to acknowledge Wang and co-worker (Xing et al., 2017) and at the same time include our mice infection data. This became even harder since our results essentially validated their findings only. Thus, in the absence of new results/insights on animal infection studies, in the revised version we focussed our attention on macrophage infection studies, omitted our data on animal experiments, and acknowledged previously reported animal infection results by Xing et al., 2017.

Reference:

Xing et al., (2017) Asymmetric structure of the dimerization domain of PhoR, a sensor kinase important for the virulence of *Mycobacterium tuberculosis*. ACS Omega, 3509-3517.

*#2: PhoPR has previously been reported to be important in Mtb response to high chloride levels, in addition to being critical for the bacterium's acidic pH response (PMID 23592993), which is presumably the reason the authors also tested 250 mM NaCl in their phosphorylation experiments? References should be added as appropriate.*

We agree. We have now included appropriate text with reference in the ‘Results’ section of the revised manuscript.

*#3: Mtb PhoR has previously been reported to have autophosphatase activity (PMID 34512602), and possession of phosphatase activity by a histidine kinase in regulation of its cognate response regulator is also known in other TCSs, including in mycobacteria (e.g. SenX3/RegX3 in mycobacteria – PMID 17526710). Acknowledgement and incorporation of these points into the manuscript, perhaps in the discussion section, will be helpful for placing the authors’ work in context.*

We thank the reviewer for the suggestion. To this effect, new text has been included with appropriate references in the ‘Discussion’ section (lines 456-458) (duplicated here):

“This is not an unexpected result as *M. tuberculosis* PhoR is known to exhibit autophosphatase activity (Waturuocha et al., 2021), and phosphatase activity of SK controlling functionality of the cognate RR has been reported for *M. smegmatis* *senX3-regX3* signalling system (Glover et al., 2007).”

References:

Waturuocha et al. (2021) A low prevalence single nucleotide polymorphism in the sensor kinase PhoR in Mycobacterium tuberculosis suppresses its autophosphatase activity and reduces pathogenic fitness: implications in evolutionary selection. Frontiers in Microbiology: doi 10.3389/ fmicb. 2021.724482

Glover et al., (2007) The two-component regulatory system *senX3-regX3* regulates phosphate dependent gene expression in *Mycobacterium smegmatis*. J. Bacteriol. 189, 5495-5503.

*#4: The concept of TCS cross-regulation has been studied extensively in other bacteria, and there has been a distinction made between "cross-talk" and "cross-regulation" (see for example PMID 18076326, 24675902). Incorporation of these concepts and comparison to results from other bacterial species would also be helpful in placing the authors' work in context.*

This is an interesting point. We again thank the reviewer for bringing this point to our attention. We have now included the following text with appropriate references in the ‘Discussion’ section of the revised manuscript.

Despite significant similarities of the conserved structural elements of TCS signalling systems, growing evidence suggests interactions between TCSs. These cross-talks, which were defined as communication between two functional pathways, are now referred to as cross-regulation requiring an activating signal (Laub and Goulian, 2007). While only a very few examples of natural cross regulations are known, reciprocal signalling between two TCSs (HssRS-HitRS) was demonstrated as an elegant example of bacterial signal cross-regulation integrating response to heme and cell envelope stress for an effective adaptation of *Bacillus anthracis* to its vertebrate host (Mike et al., 2017).

References:

Laub MT, Goulian M (2007) Specificity in two-component signal transduction pathways. Annu Rev Genet. 41, 121–145.

Mike LA et al. (2014) Two-component system cross-regulation integrates *Bacillus anthracis* response to heme and cell envelope stress. Plos Pathogens; e1004044

*#5: Details for the ChIP-qPCR method currently references a bioRxiv manuscript. Please either cite a peer-reviewed manuscript, or provide full details here.*

We have now cited a peer-reviewed paper describing the details of ChIP-qPCR methods.

*#6: Gene names should be all lowercase letters. The authors should also ensure that gene names are accurate (e.g. rv2390c, not rv2390).*

We are sorry for the inaccuracies. We have done thorough editing of the text and figures to ensure accuracy of gene names in the revised manuscript.

**Reviewer #3:**

*#1: This group has an established track record of studying the PhoP/PhoR two component system. The mechanisms of signal transduction is an important topic in the TB field and authors performed a series of well executed experiments with the proper controls. Hence, I have no concerns regarding scientific rigor and reproducibility. A key finding was demonstrating the interaction between PhoP and PrrB and the subsequent phosphorylation experiments. Overall, it is a well-written manuscript. However, they have not determined the precise biochemical mechanism whereby PhoR senses acidic pH. In fairness to the authors, this is certainly complex and work will require much more work.*

We thank this reviewer for these extremely encouraging comments.

*#2: In figure 3, the authors mention that “The data, which represent average of two biological replicates’’. The excel sheet shows average value of the two replicates. It is very difficult to conclude convincingly about the expression levels with just 2 replicates. Why not 3 replicates?*

We have now included two additional sets of data, and collectively four (4) biological replicates were considered for the final analyses. To this effect, a new heat-map has been generated and included in the revised manuscript (see Fig. 3A). Also, the relevant source data files (Tables S3-S4) reflecting the modifications have been included in the revised manuscript.

*#3: Fig. 7. The region selected for confocal imaging shows only a few Mtb bacilli and the infected cells, the authors should select a region where more infected cells can be seen with and without the lysotracker staining. Also, it should be stated how colocalization has been calculated in Fig. 7B; was performed by manual visualization?*

We have replaced the original confocal images of Fig. 7A with high-resolution images and selected regions containing more infected cells. Please see Fig. 7A of the revised manuscript.

To address the reviewer’s concern on analyses of co-localization data, this has now been explained in more detail in the legend to Figure 7 (lines 1127-1133) (duplicated here):

“Co-localization of auramine labelled mycobacterial strains with Lysotracker was investigated by visually scoring yellow and green punctas from at least 50 infected cells originating from 10 different fields of each of the biological replicates as described previously (Gutierrez et al., 2004; Chai et al., 2019). To determine percent co-localization, the number of yellow punctas were divided by the total number of punctas (yellow plus green). The results display average values from 50 infected cells (n=50) of each independent experiment with standard deviations determined from three biological repeats (***P≤ 0.001).”

References:

Gutierrez et al. (2004) Autophagy is a defense mechanism inhibiting BCG and *Mycobacterium tuberculosis* survival in infected macrophages. Cell 119, 753-66

Chai et al (2019) A *Mycobacterium tuberculosis* surface protein recruit ubiquitin to trigger host Xenophagy. Nat. Commun. 10, 1973. doi:10.1038/s41467-019-09955-8

*#4: Fig. 7B shows only the three readings (symbols), whereas, in the figure legend, it is mentioned that “% colocalization of auramine labelled strains with Lysotracker was measured in 10 different fields by counting at least 100 infected cells”, whereas in the Methods action it is mentioned that “%co-localization was determined from three biological repeats by analyses of more than 100 bacteria per sample from at least five random fields. Please clarify. Measurement of colocalization using software would be better if possible.*

We apologize for the confusion. We have modified the relevant text related to measurement of colocalization in the ‘Methods’ section of the revised manuscript.

Further, we have incorporated new text with appropriate references in the legend to Figure 7. Please see above our response to comment #3.

*#5: In figure 7 D, did the authors checked for the survival of these mice? Since it was a validation of the previously reported work of Wang et al, additional data of mice survival kinetics would have been more informative. Also, bacterial burden in extra pulmonary organs could also have been assessed.*

We have not checked for the survival of these mice. The animals were sacrificed by cervical dislocation. To enumerate lung burden of the bacilli, lungs were isolated aseptically, homogenized in sterile 1X PBS and plated on 7H11 agar plates after serially diluting the lysates.

The reviewer’s point is valid. We fully agree with the reviewer that the result suggesting importance of the *phoR* locus in *M. tuberculosis* pathogenesis *in vivo* was originally shown by Wang and co-workers (Xing et al., 2017) using a mutant strain that carried mutations around the primary phosphorylation site of the sensor. Essentially, our results on animal infection study only validated their observation, and we have not provided any additional information. Therefore, in the revised manuscript we focussed our attention towards cellular infection results and eliminated our mice infection data altogether. Our results unambiguously show that *phoR* locus plays an essential role for mycobacterial growth in macrophages.

We have explicitly acknowledged animal infection data of Xing et al., 2017 in the revised manuscript.

Please see our response to minor comment #1 of Reviewer 2.

Reference:

Xing et al., (2017) Asymmetric structure of the dimerization domain of PhoR, a sensor kinase important for the virulence of *Mycobacterium tuberculosis*. ACS Omega, 3509-3517.

*#6: Line 388-390, it is mentioned that deltaphoR showed an approximately 330-fold lower bacterial burden in mice lungs relative to WT-H37Rv, however, in Fig. 7D it looks like a 2log10 difference which is only 100-fold. Please clarify.*

This part of the results section has been deleted in the revised manuscript.

*#7: Although the authors have attempted to answer this in the Discussion (lines 455-460); why was PrrB-dependent PhoP phosphorylation not seen in deltaphoR Mtb. The given explanation is not clear. Again, can the authors explain why PhoP phosphorylation was absent in delta-phoR if PrrB can phosphorylate PhoP?*

We would like to clarify that although PhoP is phosphorylated by PrrB (Fig. 6B) and PrrB is partially phosphorylated under acidic pH (Fig. 6E), we were unable to conclusively demonstrate that PrrB phosphorylates PhoP in response to acidic conditions. In fact, we do not think PrrB functions as a sensor of acidic pH, rather we speculate that PrrB is partly phosphorylated in response to a signal that mimics altered physiology under acidic pH. Note that in the mycobacterial cell lysates while 100% of the PhoR protein was present in the phosphorylated form, only ~35% (based on the limits of detection in this assay) PrrB gets phosphorylated *in vivo* under identical acidic conditions of growth (Fig. 6E). Perhaps that is why even under acidic pH, PhoP remains unphosphorylated in a mutant strain lacking *phoR*.

This has now been explained in more detail in the ‘Discussion’ section (lines 511-525).

Please see our response to major comment #1 of Reviewer 1.

**Minor concerns:**

*Line 87; these references are incorrect, Mtb cannot respire anaerobically. The authors in the PlosOne manuscript only performed transcriptional and proteomic analysis, no respiratory assays were performed. Mtb is an obligate aerobe.*

We are sorry for the inaccuracy. We have modified relevant text and removed the incorrect references.

*Figure 2B; shouldn’t the blue square be black?*

We apologize. We have modified the column colour of the complemented mutant to maintain consistency with other figures. Accordingly, we have indicated it on the revised figure.

*Figure 3A; does the key represent fold/log difference?*

It is the fold difference(s) in gene expression that is shown in Fig. 3A

*Fig. 2B, 3B, 5C-D. The WT-H37Rv bar should have an error bar even if these panels represent the relative expression with the WT-H37Rv.*

As recommended by the reviewer, in the revised figures we have included error bars of the WT-H37Rv samples. Also, we have mentioned it in the corresponding figure legend.

*Fig. legends: please annotated the error bars and the stats used for each panel separately.*

As recommended, for each panel we have now separately indicated the error bars and mentioned the statistics in the ‘figure legends’ of the revised manuscript.

*In line 1073, “auramine labelled strains” does it mean strains or Mtb?*

In the revised manuscript we have now mentioned it as “auramine labelled mycobacterial strains”.

*Line 389, “approximately ~330-fold” please keep either approximately or ~.*

Although the above text is no longer part of the revised manuscript, we have followed the reviewer’s recommendation throughout the text.
