# Supplementary material for: Dual functioning by the PhoR sensor is a key determinant to Mycobacterium tuberculosis virulence
Source: PLoS Genet. 2023 Dec 15;19(12):e1011070. doi: 10.1371/journal.pgen.1011070 (PMC10723718; doi:10.1371/journal.pgen.1011070)
Supplement: Attachment — Submitted filename: Response to Reviewers Nov 2023.docx [file pgen.1011070.s014.docx]

Reviewer #2:

**Major comments:**

*- Fig. S2 is very confusing as currently shown, as it is unclear what the Venn diagram in Fig. S2C is showing. Are the numbers in reference to genes that are differentially regulated in ∆phoR or ∆phoP at pH 7 or at acidic pH? The reference from which the authors have taken the ∆phoP data was not done at acidic pH, so presumably this is at pH 7 (or more likely unbuffered)? Yet this figure is called out in a section of the text where the focus is on the response of the bacteria to acidic pH. The text thus does not flow, and it is unclear what this overlap of only 42 genes is supposed to mean, and how this relates to the data described previously in that paragraph. Simply taking the gene list from a different paper is likely somewhat misleading for providing a true comparison, given it is unclear that the conditions under which the RNAseq was done here versus the other paper is comparable. Can the authors please clarify. Also note the figure legend in the supplemental word file does not match that in the pdf file containing all the parts of the manuscript.*

We agree with the reviewer and understand that Fig. S2C showing a Venn diagram of either *phoR*- or *phoP*-regulated genes at pH 7.0 is somewhat irrelevant here. We are sorry for the confusion. The Venn diagram was included in the revised version to address one of the reviewer’s comments on (a) whether the differential regulation of genes is specific to PhoR and (b) what is the number of overlapping genes regulated by PhoP and PhoR.

To improve clarity on this point, we have now omitted the Venn diagram, and part of the text related to the Venn diagram. However, based on this study and a previously-published study (Solans et al., 2014), we included text describing differential regulation of genes in *ΔphoR* and *ΔphoP* under normal conditions of growth (pH 7.0) at the end of the paragraph in the ‘results’ section of the revised manuscript (duplicated below).

“Along the line, a comparison of transcriptomic data of *phoR-*mutant (reported in this study) and a previously reported *phoP*-mutant, grown under normal conditions (pH 7.0; see methods) (Solans et al., 2014) appears to suggest regulation of 157 and 152 genes (p ≤ 0.05) by PhoR and PhoP, respectively, displaying an overlap of ~42 genes.”

Finally, modified Fig. S2, figure legend and the relevant results have been adjusted accordingly in the revised manuscript.

We apologize for the discrepancy in figure legend of supplementary word file and that of the consolidated PDF containing other parts of the manuscript. We have now rectified the error.

Reference:

Solans L, Gonzalo-Asensio J, Sala C, Benjak A, Uplekar S, Rougemont J, Guilhot C, Malaga W, Martin C, Cole ST. (2014) The PhoP-dependent ncRNA Mcr7 modulates the TAT secretion system in *Mycobacterium tuberculosis*. PLoS Pathog 10:e1004183.

*- The authors now include new data in Fig. S3 for 2-3 genes that show induction at acidic pH in a ∆phoR mutant. However, no corresponding data for WT and the complemented ∆phoR mutant is shown. This should be included. The strains and RNA would appear to be in hand given the data shown in Fig. 3, so it is unclear why the authors did not include this. As noted in the original review, particularly given the apparent range in behaviour of these PhoR-independent, acidic pH-inducible genes in WT Mtb, it is important that complementation of the ∆phoR mutant phenotype be demonstrated here.*

As recommended by the reviewer, we have now included acidic pH-inducible gene expression data for both WT-H37Rv and *ΔphoR*-complemented mutant as Figs. S3B and S3C, respectively. The revised supplemental figure (Fig. S3), figure legend, and the relevant results have been adjusted accordingly in the revised manuscript.

**Minor comments:**

*- A suggestion to include Fig. S6 as part of Fig. 7, for ease of reader access to the data.*

We thank the reviewer for this suggestion. Fig. S6 has now been included as a part of Fig. 7. To this effect, modified Fig. 7, figure legend, and the relevant results have been adjusted accordingly in the revised manuscript.

*- Lines 484-486: Phrasing of this sentence is hard to understand. “Despite” does not seem to be the appropriate connecting word here.*

We totally agree. We have modified the statement in the revised manuscript (duplicated below):

“While conserved structural elements of TCS signalling systems are significantly similar, growing evidence suggests interactions between TCSs. To explore a possible non-canonical mechanism of activation of PhoP, we examined cross-talk between PhoP and other mycobacterial SKs.”
